# Delivering Prognostic News to Older People with Chronic Disease: What Format Preference and Level of Involvement in Decision Making? A Hospital Survey

**DOI:** 10.3390/healthcare11030444

**Published:** 2023-02-03

**Authors:** Ebony T. Lewis, Kathrine Hammill, Rebekah Culbert, Madeleen van der Merwe, Ashlyn Sahay, Robin Turner, Magnolia Cardona

**Affiliations:** 1 School of Population Health, University of New South Wales, Sydney 2052, Australia; 2 School of Psychology, The University of New South Wales, Sydney 2052, Australia; 3 School of Science and Health, Western Sydney University, Campbelltown 2560, Australia; 4 Occupational Therapy Services, Camden and Campbelltown Hospitals, Campbelltown 2560, Australia; 5 Institute for Evidence-Based Healthcare, Bond University, Robina 4226, Australia; 6 School of Nursing, Midwifery and Social Sciences, Central Queensland University, Mackay 4740, Australia; 7 Biostatistics Unit, Otago Medical School, University of Otago, Dunedin 9054, New Zealand; 8 EBP Professorial Unit, Gold Coast University Hospital, Southport 4215, Australia

**Keywords:** chronic disease, patient preference, decision making, prognosis, hospital, survey, older adults

## Abstract

Shared decision making near end of life is a balancing act of communicating prognosis to patients and their surrogates/families and engaging them in considering value-concordant management choices. This cross-sectional survey aimed to determine the format in which older patients with chronic illnesses would prefer to receive prognostic information on their treatment options and disease progression, and their desired level of engagement in decision making. With a 60% participation rate, 139 inpatients in two hospitals and five surrogates were presented with six hypothetical scenarios with a randomly assigned sequence: verbal and written summary, graph, table, photo, video, and pamphlet. The majority (76%) of respondents chose the traditional verbal communication of prognosis by their doctor with a written summary as a reference and to share with family; the second choice was a condition-specific pamphlet (63%). Many found the graph and photo to be distressing (36% and 42%, respectively). Most (71%) wanted to know everything about their condition trajectory, and 63% chose shared decision making rather than completely autonomous or full delegation to clinicians or family. There were no gender differentials between wanting to know it all, supporting shared decision making or the preferred format for breaking news (*p* > 0.05). Older hospitalized patients with chronic conditions are willing to discuss end-of-life issues, learn about their prognosis, and be involved in shared decision making. Innovative formats such as graphs, videos, or photos were not welcome as part of the prognostic discussion.

## 1. Introduction

Shared decision making in health is a compromise position where the clinicians cannot adopt a paternalistic attitude of prescribing without consent, nor do patients have the ‘autonomy’ of full responsibility for decisions after the transfer of information on options [1]. Shared decision making involves the commitment of clinicians to appropriately deliver sufficient relevant information and patients taking a proactive role in regard to using the information to weigh-up choices against their personal values and preferences under the guidance of the treating team [2].

A crucial step in this balancing act of shared decision making is communicating prognoses to patients and their surrogates or relatives [3]. Published surveys in the 1990s indicated a patient’s preference for receiving information about their diseases rather than an inclination to get involved in decision making. Poor education, male gender, older age, and religious/cultural background were associated with a less involved role in clinical decision making [4]. The purpose of delivering accurate information is to enable patients and close relatives to participate in setting goals of care [5], incorporating non-medical and medical issues that matter to them, as well as treating goals and improving quality of care [6]. A common problem patients report is either not being told their prognosis or misunderstanding the status of their disease, the aim of their treatment and their prognosis [7,8,9].

The current recommendations for oncologists is to use a variety of techniques in the communication with patients and to make sure the information delivery has been successful [10,11,12]. The medical literature provides limited to no evidence of the best ways of presenting prognostic information to optimize understanding, psychological adjustment, and decision making within the elderly patient population with other multiple chronic life-threatening non-malignant conditions. One of the major challenges is finding the most appropriate way to get information across to patients. Some minimum components of this communication are “how long patients will survive” and “how good” their quality of life will be [13].

Recently, evidence that patients and their families want varied levels of involvement is growing [14,15,16,17,18,19]. Indeed, guidelines for less paternalistic and more patient-centred involvement in decisions are recommended [20] as an integral part of routine care after prognosis is known. This includes the ascertainment of patient preferences and values and conveying information in a clear and sensitive manner [21,22]. This study aimed to explore more in-depth the patient and surrogate perspectives on their preferences and involvement in the prognostic communication for chronic illness.

### Objectives

Our primary goal was to understand the prognostic information preferences of older patients and their surrogates about chronic illness. The specific objectives were as follows:

- To determine the format in which hospital patients with advanced age and chronic illness and/or their surrogates prefer to receive information on prognosis in the hypothetical case that they were the recipients of this information.
- To describe the relationship between prognostic information preference and particular patient factors including values, demographics, and clinical characteristics.
- To ascertain the depth of the prognostic knowledge that patients and/or their surrogates would be interested in obtaining in the hypothetical case that they were the recipients of this sensitive news (e.g., survival rates, harms and benefits of each treatment option, cost, quality of life with and without treatment, etc.).
- To ascertain patient or surrogate perspective on the preferred role and extent of involvement in health/treatment decisions following poor prognostic news.

## 2. Materials and Methods

A cross-sectional mixed methods study conducted by two interviewers in two hospitals over six months to achieve the required sample size included a quantitative assessment using a researcher-administered survey questionnaire containing closed-ended questions and Likert scales, and a qualitative inquiry via open-ended questions. The questionnaire design was suggested by mutual agreement between the clinicians and epidemiologist in the research team about the types of visual aids and including a variety of conditions in anticipation of the case mix of our future respondents. The written summary and table were custom-made; the photos, video, and pamphlet were taken from the Internet. We ran several drafts and a brief consultation with university colleagues. The scenarios were refined after piloting with older chronically ill consumer advisors.

### 2.1. Participating Sites

This research was completed at two medium-to-small-size (<310 beds) teaching hospitals in Sydney, Australia, between May and November 2019. Medical, surgical, chronic, acute, and rehabilitative wards were selected to ensure that a variety of conditions were represented.

### 2.2. Sample Selection

Primary data collection using the quota sampling approach was used as an accepted alternative to random sampling of individuals [23]. Instead, random sampling of weekdays was generated to select the days that recruitment took place to attain a sample representative of the hospital clientele during the study period. To ensure the inclusion of both sexes, and different age groups and diagnostic categories in the patient population, a stratified quota sample was predefined with mutually exclusive subgroups. That is, on each survey day, interviewers (data collectors) recruited eligible patients present on the ward until the required age group and sex quotas predetermined by the researchers were filled. Selection of participants from the eligibility pool was based on date of admission, i.e., those with longest length of stay were recruited first on survey days and most recently admitted patients were recruited last on the day.

### 2.3. Sample Size Estimation

Based on the availability of the data collectors (three days per week during weekdays), we calculated that it was feasible to recruit 5 participants per day, giving an anticipated total sample of 240 participants (120 men and 120 women) recruited in the two hospitals over four months considering the case mix and weekly intake on selected wards. This was estimated to provide 80% power to detect a difference of 15% in the top prognostic preference modality between men and women at the 0.05 significance level as per PS Power and Sample Size Calculator software version 3.1.6, developed by Vanderbilt School of Medicine.

### 2.4. Eligibility and Exclusion Criteria

Our focus was not on target diagnoses but on target hospital wards. This is because our intention was to pilot tailored ways of delivering prognostic information in general, rather than for specific diseases. Older patients (aged ≥ 70 years) were potentially eligible to participate if admitted to hospital for chronic or acute management of their illness in the following units: respiratory, general medicine, cardiology, geriatrics/aged care, rehabilitation, orthopaedics, medical transition unit, dialysis unit, and chemotherapy/radiotherapy same-day units. The surrogate was invited to be a survey respondent if the patient was eligible but unable to give written consent.

Exclusion criteria: Patients who were imminently dying, or unconscious, were not included in the study. Likewise, patients who were not admitted or were in the emergency department, and those who were unable to communicate in English unless they had an interpreter, or who had cognitive impairment unless a consenting surrogate was available, were not included in the study.

### 2.5. Recruitment

On recruitment days, the patient eligibility pool from the targeted wards was determined by the research interviewers via Power Chart (hospital electronic medical record) and by identifying the potentially eligible patients with the medical registrars. Potential participants were prioritized by imminency of date of discharge. Once eligibility was confirmed, patients were approached by a research team member who was not involved in their care, with verbal information, the opportunity to ask for clarifications, and a participant information statement. They were given time to read the document and consider participation, and were encouraged to discuss their involvement with their family prior to their acceptance. Surrogate decision makers were invited to participate on their loved one’s behalf if they were present at the time of recruitment. Participants were able to withdraw consent once the survey had commenced if they changed their mind or became distressed during the data collection. This study received ethical approval from the South Western Sydney Local Health District Human Research Ethics Committee [#2019/ETH00112]

### 2.6. Survey Administration and Questionnaire Contents

The survey was developed for the purpose of this study and previously piloted for feasibility and comprehension [24]. The feasibility markers previously reported were (1) ability to recruit older hospitalized patients; (2) patient/surrogate acceptance of an instrument with sensitive questions such as prognostic preference and desired level of disclosure without dropping out of the interview; and (3) acceptability of discussion of different visual formats of breaking news among patients with life-limiting illnesses.

The researcher-administered survey interview lasted for approximately 20 min using an iPad to present visual material for participant convenience, and was conducted by nominated members of the research team without any third party involvement to minimize biases. As previously reported [24], administration followed a computer-generated random list assignment of the scenario sequence by an external statistician. The list of random numbers was followed in consecutive order. Respondents could be the patients themselves or their surrogate could be if present at the time of interview. In addition to demographic and clinical information (reason for admission, comorbidities), the priorities for health and well-being (up to 3 pre-coded answers), interest in participating in decision making if given poor prognostic news (up to 3 pre-coded answers), and preference on formats to receive poor prognostic news were collected. The specific survey contents is provided in Supplementary Materials. In brief, questions included:

(1) What would you say is most important to your health and well-being? (Select maximum of three options.)
(2) I want you to think about a situation when you would need to make an important decision about treatment for your [insert patient’s chronic condition]. Would you prefer to (select maximum of three options)?
(3) How much information would you like to know about what brought you to hospital and whether it will get better or worse in the next few months? (Select maximum of one option and document a free text response for reason for preference.)
(4) What are the types of things that you would like to know about your condition? (Select all options relevant.)
(5) If you were to receive news about how your current health issues will progress in the future, how would you prefer to receive the news? (Select maximum of one option.)
(6) Six hypothetical formats were than presented to participants. For each hypothetical scenario as shown in Box 1, response options were given for each format selected by respondents from a 5-point Likert scale ranging from “like it a great deal” to “dislike it a great deal”, and free text responses could be documented for reason for preference.

Box 1 **Hypothetical scenario options for preference on formats to receive poor prognostic news.**

(1) Verbal information from the doctor with a summary of the condition, treatment options, and progression?
(2) Verbal information from the doctor plus graphs explaining the survival rates of a condition?
(3) Verbal information from the doctor plus tables containing numbers and percentages of survival rates of a condition?
(4) Verbal information from the doctor plus a photo of patient having a procedure completed?
(5) Verbal information from the doctor plus a pamphlet on a condition and its treatment options?
(6) Verbal information from the doctor plus a link to a video of what a procedure would entail?

### 2.7. Data Analysis and Synthesis

Descriptive statistics (absolute and relative frequencies) were used to present survey responses. Results are presented in tables. Demographic differentials were only analysed when a response was not evidently a vast majority (e.g., if 90% of participants agreed on something, we did not explore male–female differentials). Cross-tabulations by age, gender, and education were set for those not wanting involvement in decision making and the chi-square was used to investigate associations. This was conducted to better understand the patient’s perspective and to identify possible points for intervention in the future. ETL independently conducted qualitative analysis. Data responses to the open-ended free text questions pertaining to the level of prognostic information the participant would like to know and the reason for preference to liking/disliking each hypothetical format were subjected to thematic analysis [25] and managed using NVivo software (version 12 QSR, International Pty Melbourne, Victoria, Australia). Trustworthiness of the interpretations was enhanced by providing direct but deidentified quotes from participants.

## 3. Results

### 3.1. Participant Profile

**Figure 1:**
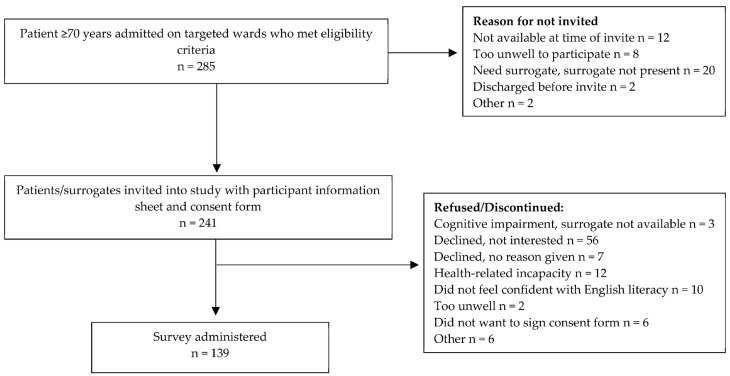
Flow chart of the recruitment procedure.

A total of 285 patients were potentially eligible, of which 241 (85%) were invited as the rest were either too unwell to participate, did not have a surrogate present, or were not on the ward at the time of recruitment (see Figure 1). Of the 241 invited, 139 people (60% of the expected sample size, 96% of patients, and 4% of patients with surrogates present) from the general medicine, respiratory, aged care, and oncology wards responded to the survey.

The demographic profile was relatively homogeneous (Table 1), with all being English-speaking, all with some form of Christian religious affiliation, two thirds being relatively uneducated, over half of them being aged 80+ years, and most having chronic diseases.

### 3.2. What Is Important to Hospitalized Older People?

To the vast majority, family, loved ones, friends, and pets were the most important contributors to their health and well-being, followed by their principles and beliefs, and their mental and physical capacity (Table 2A).

Patients were then presented with a hypothetical scenario where they were told by a clinician that their condition would deteriorate over the next few months. They were consulted about their wishes for level of knowledge and participation in decision making about their condition.

### 3.3. Making Important Treatment Decisions

In relation to preference for participation in decision making, they were asked about the issues that they considered when needing to make an important decision about the treatment of their chronic condition; half of the respondents were interested in sharing the responsibility with both the clinician and their family, and a quarter wanted to make the decision by themselves. Fewer participants preferred to make the decision only with the doctor, and a minority chose letting the doctor or family decide on their behalf (Table 2B).

**Table 2:** Domains on well-being and treatment decisions for all respondents (N = 139) *.

(A) What Is Most Important to Your Health and Well-Being?	N (%) Selected This Answer	(B) Aspects you Consider When Needing to Make an Important Decision about Treatment	N (%) Selected This Answer
Relationships—with your family, loved ones, friends, and pets	109 (78)	Sharing the responsibility of the decision making with your doctor and your family regarding which treatment is best for you	69 (50)
Principles—the things you believe in	62 (45)	Making a decision after serious consideration of your doctor’s opinions	30 (22)
Abilities—your physical or mental capacity or skills	57 (41)	Having the final choice about your treatment	27 (19)
Emotions—such as your feelings and mood	54 (39)	Sharing the responsibility of the decision making only with your doctor regarding which treatment is best for you	19 (14)
Activities—such as work, hobbies, volunteering	53 (39)	Letting your doctor decide which treatment is best on your behalf	10 (7)
Possessions—your objects and belongings which have personal meaning	10 (7)	Letting your family decide with your doctor on your behalf	10 (7)

* More than one response allowed for each question.

No statistically significant demographic association was found between those preferring to make the decision alone or delegate it to family or their doctor, and those welcoming shared decision making (by gender χ^2^ 0.092, *p* = 0.761; by age group χ^2^ 2.699, *p* = 0.440; by higher/lower education χ^2^ 0.312, *p* = 0.578; by living alone χ^2^ 0.444, *p* = 0.505; or having ≥2 comorbidities χ^2^ 0.305, *p* = 0.371).

### 3.4. Preference for Depth of Knowledge on Prognostic News

After being asked how much information they would like to know about what brought them or their loved one to hospital and whether it will get better or worse in the next few months, a vast majority wanted to know everything and a slim minority preferred not to know anything (Table 3A). Bivariate analysis did not uncover any statistically significant factors associated with not wanting to know everything (by gender χ^2^ 2.256, *p* = 0.133; by age group χ^2^ 2.678, *p* = 0.444; by higher/lower education χ^2^ 0.162, *p* = 0.687; by living alone χ^2^ 0.017, *p* = 0.897; or ≥2 comorbidities χ^2^ 0.39, *p* = 0.499).

#### 3.4.1. Reason for Desired Depth of Knowledge

Respondents stated various reasons for selecting their desired depth of prognostic information. For the majority who stated a preference for receiving prognostic information, the main theme was knowing the trajectory of their condition as it was perceived to help both the patient and their relatives be better prepared for the future:

“Well I think if you are going to die, I would want to know and I would want to be prepared. I would want my family to know. To know when your last sunset is going to be”.

(male, 72 years)

Prognostic disclosure by the clinician was viewed as integral to decision making in another important theme. In this sense, the information provided could assist the individual in making informed decisions about their treatment options and could provide a person with a sense of control over their own health:

“Helps you to make an informed discussion and make choices about if you want something to happen”.

(female, 82 years)

Knowing what to expect was consistently said to be a driving factor for wanting to know prognostic information even if the prognosis was poor. This theme was perceived to reduce negative emotions, as the person would know the trajectory of their condition: “Personally, I would be more stressed if I didn’t know the direction my health would go. It’s better to know everything” (male, 75 years).

However, in contrast, for one participant, receiving poor prognostic news was thought to take away hope: “I would only want to know enough to give me hope. I don’t want to know information that would make me lose hope” (male, 80 years).

While truth disclosure of the prognosis was viewed as a patient ‘right’, for a small minority, there was a preference not to know all of the information and instead the family members were the preferred recipients: “I wouldn’t want to go into all the details, but the kids want to know all the details” (female, 83 years).

#### 3.4.2. Types of Prognostic Information

When asked what types of prognostic information respondents would like to know about the condition in question, the most sought-after details were the benefits of treatment. This was followed by more than half wanting to know treatment side effects, chances of cure, and treatment impact on quality of life. Less than half were also interested in survival time, cost of treatment, and impact on family (Table 3B).

### 3.5. Most Preferred Formats If Delivered Poor Prognostic News

The majority of respondents preferred to receive poor prognostic news in the format of a doctor providing verbal news supplemented with a written summary on their condition (76%), and being told with a pamphlet outlining the condition, treatment options, and progression was desired by 63% of respondents (Table 4).

#### Reasons for Preferred Formats

The theme “valuable reference” emerged prominently. The reasons for liking written information in the form of a summary and pamphlet were for the convenience of a document to refer back to after the consultation, as overwhelming news could cloud their understanding or recall of the key points mentioned by the clinicians. “This would be great, as often information just goes flying past your head. If you can understand it in the first place. And then trying to remember it and get it right is difficult” (male, 85 years). For others, written information was helpful to understand the course of the illness and its trajectory: “Want to know everything and this could help. I would particularly want to know what to expect at the end” (male, 82 years).

The theme “balance to prevent emotional burden” was obvious for others. Respondents commented that having a written summary option provided an opportunity to fill information gaps when verbal communication was hurried or unsatisfactory. “There are a lot of things you don’t know and you may not think to ask. This might help cover it all” (female, 83 years). A small number of respondents did, however, comment that verbal information accompanied by a written summary should be balanced, as information in the form of written communication may cause a patient to worry if excessive: “Too much information would make me worry, when my time comes, I just want them to talk to me” (female, 74 years).

### 3.6. Most Disliked Formats If Delivered Poor Prognostic News

By contrast, the most disliked format (46.7% of all respondents) for delivering prognostic news was being provided with a photo of a patient having a treatment, which 42.4% of all respondents found distressing. “Cognitive dissonance” appeared to be a common theme. While many respondents found the photo distressing, it was acknowledged by a small minority that a photo may provide a level of understanding as to what could be expected during a treatment: “It may be distressing, but I’d still want to see it so I have an idea” (female, 71 years). Many respondents stated a preference to not to look at the photo as they found it upsetting and worrying or considered it irrelevant to their situation: “May not represent your own experience and it makes you feel sad for the people in the pictures and makes you feel defeatist” (female, 76 years).

For others, there was a strong preference to go ahead with the procedures without viewing visual material prior to the procedures, or would prefer to be in denial: “I’d prefer not know. I don’t think I’d like it. Otherwise I’d be thinking about what I saw in that photo and I’d prefer not to know what they are going to do” (female, 75 years). Likewise, the graph explaining the survival rates of a condition was disliked by 33% of respondents, and 36% of all respondents also found it distressing. Females were twice as likely to dislike the graph than males (45% vs. 25%, *p* = 0.0426).

For those respondents who disliked the graph format, most of them reported that they would find this format confusing and worrying as they would not know what it meant in general or meant for their particular situation: “I would have difficulty understanding. I don’t really want to know, it would make it hard for me to think positive” (male, 94 years). Others did not believe in their accuracy: “It’s a waste of time, they are never accurate” (female, 70 years) (Table 5).

## 4. Discussion

In this survey, we sought to understand the preferred format for receiving prognostic information, the depth of prognostic information, and the desired level of engagement in the decision-making processes of older hospitalized patients with chronic illnesses. We found that the majority of participants wanted detailed prognostic information about their condition when presented with a hypothetical case, with verbal information from the doctor accompanied by a written summary being desired over other formats for communicating prognostic news.

### 4.1. Summary of Main Findings

In our study, the vast majority of participants stated a preference for full prognostic disclosure from their treating team if their condition was to worsen, with only a small minority preferring not to know anything. This is consistent with previous research focused on the levels of prognostic disclosure of oncology patients [26,27,28,29]. While we found no statistically significant association between gender, age group, education level, living arrangements, or having ≥2 comorbidities for those who did not want to know anything about a poor prognosis, others have found that patients with higher education levels and those who are married [30] and of a younger age [28] are more likely to want full prognostic information. Our small sample size might have been underpowered to confirm these associations.

Most participants in our study, when presented with options on different delivery formats for receiving poor prognostic news, preferred verbal news by the doctor complemented with a written summary of their condition for treatment options and progression, while graphs and photos were the least preferred formats. Having a written summary was viewed as being important, as receiving poor prognostic news was seen as highly emotive and the person may not be able to process all of the information during the discussion. Respondents stated that access to a written document allows the person to revisit information at a later time, particularly if the delivery of the prognosis is rushed or unsatisfactory.

Participants in our study preferred decision making regarding treatment to be a collaborative process with the family and treating team. Nonetheless, approximately a quarter of the participants indicated the need to be exclusive decision makers regarding their treatment, while a small minority indicated only the doctor or family should make the decision. This highlights the diversity in the subjective nature of patient preferences for prognostic communication and decision-making choices. Importantly, patient beliefs and capabilities have also been identified in influencing the desired level of involvement in decisions about their own health. Patients may feel that they do not have the necessary skills to be included in the decision-making process, whereas others may have the confidence to play an active role due to past health experiences [31]. These findings re-enforce to clinicians the importance of taking the time to explain and elicit patient preferences when engaging in shared decision making.

This study indicated that regardless of the communication mediums used for the delivery of prognostic information, older adults within the hospitalized setting valued having friends, family, loved ones, and pets as important contributors to maintaining health and well-being.

### 4.2. Comparisons in the Context of the Literature

Delivering prognostic information is an essential skill for all doctors; however, sharing serious information is often complex and a difficult responsibility in the practice of medicine. The delivery of this communication can impact how a patient may perceive the information provided and their level of involvement in the decision-making process regarding their health management [31,32,33].

Similarly to our survey respondents’ reluctance to know survival time (33.1%), a larger survey of a 65+-year-old ambulatory community sample in the US presented with the hypothetical scenario of a life-limiting illness in 2016 revealed that almost two thirds of respondents (59%) did not want to discuss expected survival time. Most (88%) of them also preferred the doctor not to discuss this with family or friends [19]. While they also did not find gender differentials for this preference, the people more open to end-of-life discussions were those with life-threatening illnesses [19]. Yet, our Australian sample was more supportive of shared decision making (63.3%) than the US respondents (37.5% combined wanted either shared decision making or to leave decisions to their doctor). These differences may be related to the cultural make-up of the sample or other health system interventions to raise awareness or encourage consumer engagement.

Contrary to our findings of a lack of gender differentials in prognostic preferences, a study of 400 community retirees aged 60+ years in Brazil also using a hypothetical question found that women were less inclined than men (68% vs. 83%, respectively) to want information about having limited life expectancy (OR = 0.446: 95% CI: 0.269–0.738) [34]. Furthermore, our hospital sample had a lower interest in life expectancy (33%) or symptom progression (45%) than the Brazilian community-dwelling older adults (74% and 89%, respectively) [34]. This may indicate that the hypothetical scenarios may trigger a different perception of priorities if respondents are actually ill and hospitalized rather than if they are ambulant.

A qualitative consultation in the US asked 25 older adults hospitalized for injuries and their caregivers about their preferences for prognostic information at one year, including mortality [35]. Results revealed that caregivers were far more receptive to the news than the patients themselves (73% vs. 56%, respectively), with indications that some were resigned to poor outcomes due to advanced age and the sequela of injuries. However, even these acutely ill patients appeared to be more interested in survival information than the chronically ill inpatients in our study [35]. Cultural differences and a lack of preparedness for the inevitable due to chronicity of our participants may explain the differences.

Clinicians require support, tools, and training to build the empathetic approach needed to deliver prognostic news, especially when the news is not favourable [32]. A Polish study by Adelekan and colleagues [36] indicates the use of technology as a new medium that may assist with the delivery of diagnostic news; however, the older age group may be a factor in the uptake of technology-based mediums. Similarly, an earlier study highlights that using other formats to deliver a cancer diagnosis was favoured by a younger demographic compared to older adults, as they preferred an in-person approach [37]. While this study was specific to cancer patients, it is also aligned with the outcomes of our study where the majority of older hospitalized adults with chronic illnesses preferred visual aids the least when receiving prognostic news.

### 4.3. Implications for Practice

In this age of personalized healthcare and readily available online answers to health questions, the role of patients in their medical decisions is on the rise and patients are becoming more involved in their own healthcare. However, the specific sub-component of their prognosis that patients want to know may vary by country, current clinical acuity, setting (community vs. hospital), or other factors not explored in our study. The reasons for avoiding the life expectancy news were not explored in our study, but others have reported either scepticism in predictions or fear of psychological impact [19,38]. Clinicians will need to be better prepared to suit the needs and requests of patients/families by disclosing prognostic information in the consumer’s best preferred format, content, and depth.

The findings of this study from a sample of participants in a real-life clinical setting hope to provide clinicians with more confidence in communicating poor prognostic news which is more sensitive and acceptable to recipients (patients and surrogates), incorporating their values and preferences. The delivery of poor prognostic news aligned with the patient/surrogate preferred format and depth of prognostic information in this age group may assist in the initiation of end-of-life conversations with patients, reduce conflict, and empower both parties to agree on the goals of care. Eventually, it is hoped that these visual or verbal aids will be an integral part of the routine care of advanced chronic illnesses in and out of acute hospitals.

### 4.4. Strengths and Limitations of the Study

While this is one of the few studies addressing the sensitive issue of prognostic awareness and preference for older hospitalized patients, we did not reach the anticipated sample size due to time constraints, refusals, staff turnovers, and the social distancing restrictions at the start of the pandemic. Recruitment in diverse wards somewhat contributed to enhancing the generalizability of the findings to the target population, but sub-analysis by ward or diagnosis was not possible due to small numbers; thus, we confined the analysis of the clinical picture to the presence of two or more chronic comorbidities. It is possible that if the hypothetical scenario coincided with the respondent’s own illness, accuracy may have differed in responses to non-relatable scenarios. Our intention was to examine the tailored ways of delivering prognostic information in general, rather than for specific diseases, so participants were advised that the prognostic information shown in the scenarios was not personalised to their present situation, and we had no way of measuring the accuracy of responses. As we had limited time and resources for this study, we used quota sampling. While quota sampling ensured the sample was representative of gender and age groups, it is possible that other characteristics may have been disproportionately represented [23,39]. Our hospital sample was not ethnically or culturally diverse, but it was representative of the geographic area under study.

### 4.5. Future Research Directions

An additional valuable investigation in a future larger sample could be the analysis of factors beyond age associations with preference for non-shared decision making, such as the one in five patients in our study who wanted to either delegate responsibility to the doctor or their family without patient involvement, or preferred to make the decision alone. Additionally, replicating the survey in a younger age group with terminal illnesses may yield different preferences for both delivery formats and level of involvement in decision making. Future research could also explore changes in patient prognostic information preferences over time, as preferences may change over the course of an illness as the end approaches.

## 5. Conclusions

Older hospitalized patients with chronic comorbidities and their families were willing to learn about their prognosis, preferred verbal disclosure supplemented with a written summary, and had varied preferences for decision making, predominantly shared with the doctor and family, or with the doctor only. Relationships with family and friends were their priority domain for health and well-being, and their preferences for information about their condition were mostly treatment benefits and risks. We confirmed that it is feasible to conduct this survey regarding the sensitive topic of preferences of older patients and their surrogates for formats and contents of prognostic information in a hospital setting and investigate the context and extent to which this information should be delivered. This should encourage clinicians to pursue the end-of-life care discussion in routine care.

The survey also provided an opportunity for patients and families to share their perceptions of what level of prognostic information and consumer involvement they would be prepared to have. It is anticipated that this will help healthcare providers to improve the ways they currently break bad news and help service managers in facilitating these resources. In designing decision aids, algorithms, or conversation guides to involve older people in the future, it might not be wise to invest in additional visual aids such as graphs, videos, or relevant photos, as they are not welcome by this age group.

## Figures and Tables

**Table 1:** Participant characteristics (N = 139).

Demographic and Clinical Characteristics		N (%)
Sex	Male	61 (43)
	Female	78 (56)
Surrogate present	Yes	5 (4)
Country of birth	Australia	105 (75)
	UK	18 (13)
	Elsewhere	16 (12)
Religion	Anglican or Catholic	84 (61)
	Other Christian	54 (39)
Age group	70–74 years	24 (17)
	75–79 years	41 (30)
	80–84 years	35 (25)
	>85 years	38 (28)
Household structure	Living with partner/spouse or child	75 (54)
	Living alone	52 (37)
	Extended family or residential aged care	13 (9)
Education	Primary or no formal	9 (7)
	High school	78 (56)
	Trade or technical training	37 (27)
	Tertiary degree	14 (10)
Comorbidities	At least one chronic illness	123 (88)
	At least two comorbidities	83 (60)
Reasons for admission	Respiratory	31 (22)
	Fall	29 (21)
	Pain	19 (14)
	Other causes: most frequent admission diagnoses being heart attack, chronic pulmonary disease, stroke, chronic heart failure, kidney disease, and other liver conditions	60 (43)

**Table 3:** Depth and type of prognostic information that survey respondents wanted to know.

(A) How Much Information Would You Like to Know about Whether Your Condition Will Worsen?	N (%) Selected This Answer	(B) What Types of Things Would You Like to Know about Your Condition? *	N (%) Selected This Answer
I would want to know everything	98 (71)	Benefits of treatment	100 (72)
I would want to know some information	35 (25)	Treatment risks or side effects	85 (61)
I would not want to know anything	5 (4)	Chances of cure	81 (58)
		Impact of treatment on quality of life	77 (55)
		Explanation of how the disease will progress	62 (45)
		Expected survival time	46 (33)
		Cost of treatment/management	46 (33)
		Impact of treatment/management on family or carers	44 (32)
		Changes in complications	36 (26)
		Other treatment alternatives	31 (22)
		Prefer not to say	2 (1)

* More than one response allowed for each question.

**Table 4:** Reasons for liking the top preferences of communication formats for prognostic news.

Doctor Verbal News with a Written Summary Reasons and N Respondents (Out of 105 People Who Liked This Format)		Doctor Verbal News with Pamphlet on a Condition Reasons and N Respondents (Out of 87 People Who Liked This Format)	
Reason	N (%)	Reason	N (%)
Reference to check and remember	40 (38)	Reference to check and remember	28 (32)
Understand course of illness	12 (11)	Understand course of illness	25 (29)
Fill information/communication gaps	10 (10)	Can be shared and clarifies for family	12 (14)
Offers time to digest news	9 (9)	Language comprehension	6 (7)
Can be shared and clarifies for family	8 (8)	Informs treatment decisions	2 (2)
Comprehend illness and terminology	6 (6)	Offers time to digest news	1 (1)
Informs treatment decisions	4 (4)	Fills information/communication gaps	1(1)
Reduces apprehension	4 (4)	Helpful/unspecified	10 (11)
Language comprehension	2 (2)	No reason	2 (2)
Helpful/unspecified	10 (10)		
Other various reasons or no reason	3 (3)		

**Table 5:** Reasons for disliking the top communication formats for prognostic news.

Doctor Verbal News with Photo of a Patient Undergoing Treatment (Out of 65 People Who Disliked This Format)		Doctor Verbal News with Graph Explaining Survival Rates of a Condition (Out of 50 People Who Disliked This Format)	
Reason	N (%)	Reason	N (%)
Prefers not to see the photo	14 (22)	Cannot understand graphs	19 (38)
Finds photo upsetting	10 (15)	Leads to negative thinking	6 (12)
Considers photo may be irrelevant	7 (11)	Does not help make decisions	4 (8)
Best to just have the procedure	7 (11)	Confronting, depressing, confusing	4 (8)
It would worry them about the future	6 (9)	Prefers to read/hear/live in the present	4 (8)
Finds photo frightening	5 (8)	Sceptic—graphs are inaccurate	3 (6)
Prefers just to be told	5 (8)	Information would worry them	3 (6)
Prefers not to know what is coming	4 (6)	Unhelpful/unspecified	5 (10)
Too much information/already knows	2 (3)		
Previous experience/not having procedures	2 (3)		
Other mixed reasons	3 (5)		

## Data Availability

Data supporting reported results are not available for sharing due to ethical approval conditions. The corresponding author can be contacted for any request for processed data.

## Supplementary Materials

The following supporting information can be downloaded at: https://www.mdpi.com/article/10.3390/healthcare11030444/s1, Survey questionnaire content.
