# Supplementary material for: Delivering Prognostic News to Older People with Chronic Disease: What Format Preference and Level of Involvement in Decision Making? A Hospital Survey
Source: Healthcare (Basel). 2023 Feb 3;11(3):444. doi: 10.3390/healthcare11030444 (PMC9913994; doi:10.3390/healthcare11030444)
Supplement: Supplementary file 1 [file healthcare-11-00444-s001.zip › healthcare-2091488-supplementary.pdf]

Supplementary Materials

Survey questionnaire contents

Note: For surrogate respondents, wording of questions was modified for example 'your health' was modified to read 'your loved one's health'

Survey question and instructions	Survey response options
Participant health and socio-demographic characteristics<br><br>*(If surrogate is the participant, record the surrogate's level of education, ethnicity, and religion)*	Age, gender, reason for hospitalization, admitting ward, life-limiting condition, time since diagnosis, language spoken at home, level of education, ethnicity, religion, country of birth, living arrangements, post code, etc.
Survey completed with	☐ Patient<br>☐ Surrogate<br>☐ Patient and Surrogate
What would you say is most important to your health and well-being?<br><br>*(Select minimum of 1 and maximum of 3 options)*	☐ Principles - the things you believe in<br>☐ Relationships - with your family, loved ones, friend, and pets<br>☐ Emotions - such as your feelings and mood<br>☐ Activities - such as work, hobbies, volunteering<br>☐ Abilities - your physical or mental capacity or skills<br>☐ Possessions - your objects and belongings which what personal meaning<br>☐ Prefer not to say
I want you to think about a situation when you would need to make an important decision about treatment for your [insert patient's chronic condition]. Would you prefer to:<br><br>*(Select maximum of 3 options)*	☐ Have the final choice about your treatment<br>☐ Make a decision after serious consideration your doctor's opinions<br>☐ Share the responsibility of the decision making with your doctor regarding which treatment is best for you<br>☐ Share the responsibility of the decision making with your doctor and your family regarding which treatment is best for you<br>☐ Let your doctor decide which treatment is best on your behalf<br>☐ Let your family decide with your doctor on your behalf<br>☐ Prefer not to say
How much information would you like to know about what brought you to hospital and whether it will get better or worse in the next few months? [Ask participant to explain their response by asking them why they chose that answer. Please document reason]<br><br>*(Select maximum of 1 options)*	☐ I would want to know everything<br>*Reason*____________________<br>☐ I would want to know some information<br>*Reason*____________________<br>☐ I would not want to know anything<br>*Reason*____________________<br>☐ Prefer not to say
What are the types of things that you would like to know about your condition? [Do not prompt participant at first. Write down what the participant says or tick as many options stated as are relevant]<br><br>*(Select all options stated that are relevant)*	☐ Participant Response____________<br>☐ Benefits of treatment<br>☐ Treatment risks or side effects<br>☐ Chances of cure<br>☐ Impact of treatment on quality of life<br>☐ Changes of complications<br>☐ Impact of treatment/management on family or carers<br>☐ Cost of treatment/management

	☐ Other treatment alternatives<br>☐ Explanation of how the disease will progress<br>☐ Expected survival time<br>☐ Prefer not to say
If you were to receive news about how your current health issues will progress in the future, how would you prefer to receive the news?<br><br>*(Select maximum of 1 options)*	☐ Verbal information from the doctor<br>☐ Verbal information from the doctor plus graphs outlining my condition, treatment options and progression<br>☐ Verbal information from the doctor plus tables outlining my condition, treatment options and progression<br>☐ Verbal information from the doctor plus pictures of the treatment I would have to undergo in the future<br>☐ Verbal information from the doctor plus a pamphlet outlining my condition, treatment options and progression<br>☐ Verbal information from the doctor plus a link to a website outlining my condition, treatment options and progression<br>☐ Prefer not to say
Show participant card with **hypothetical scenario options*** Explain to participant "That the following examples that I will show you are only hypothetical. They do not apply to your specific scenario"<br><br>Hypothetical Scenario Order Sequence as per Randomisation<br>Verbal Information from doctor with<br>▪Written summary<br>▪Graphs<br>▪Table with numbers<br>▪Picture of patient procedure<br>▪Video<br>▪Pamphlet<br>*For each hypothetical scenario participant to specify their preference (Likert scale); reason for preference (free text); distress scale (Likert scale)*	**1.** On a scale of 1 to 5, how much would you like to be shown [insert hypothetical scenario]<br>Dislikes it strongly Likes it strongly<br><br>____1______2_______3_______4_______5_______<br><br>**2.** Please comment on your reason for this preference<br><br>*Reason* ___________________________________<br><br>**3.** On a scale of 1 to 5, how distressing did you find [insert hypothetical scenario]<br>Not at all distressing Very distressing<br><br>____1______2_______3_______4_______5_______

*Hypothetical scenario options presented to participant

Verbal information from the doctor and a summary of a condition and treatment options

Visit Summary

Key points we discussed today:

- Your diagnosis is a chronic lung condition
- Your oxygen saturation is below 90% most of the day
- Your lung capacity is severely reduced to 50% of normal volume in an adult

Treatment options for you and outlook:

- A physiotherapist can teach you techniques to reduce your breathing effort.
- This won't extend your life, but it will help enhance your quality of life.
- An occupational therapist can help you to continue to keep doing the everyday activities you wish to continue.
- In the end-stage of your diagnosis you'll likely need supplemental oxygen to breathe and you may not be able to complete activities of daily living.
- If your lung capacity is less than 35% you may not survive beyond four years after this result.

Instructions:

- Use the nicotine patches to help you stay off smoking
- Try to maintain your weight; do not lose weight because you will become frailer and less able to mobilise yourself.
- If your shortness of breath is hard to manage or you cannot walk too far in six minutes this means your condition has progressed, and you may benefit from additional palliative or hospice care.

Contact Nurse XXX or Doctor YYY if you have any further questions

______________________ ___________

Treating doctor Date

Graph explaining the survival rates of a condition

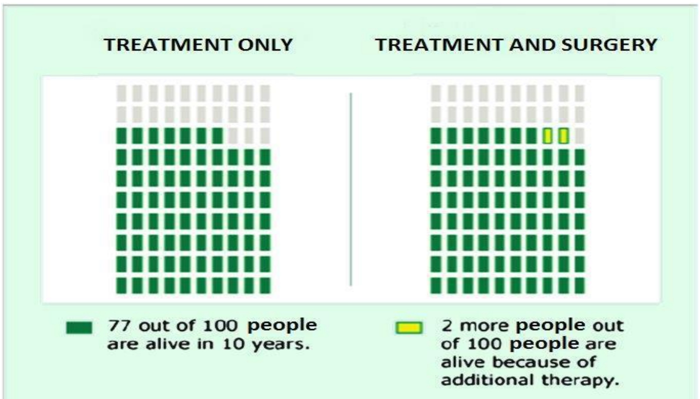

Table containing numbers and percentages of survival rates of a condition

	3-year survival	5-year survival	10-year survival
Chemotherapy	99 %	97 %	91 %
Radiotherapy	95 %	89 %	67 %
Surgery	89 %	81 %	50 %
Immunotherapy	70 %	57 %	39 %

Picture of a patient having a procedure completed

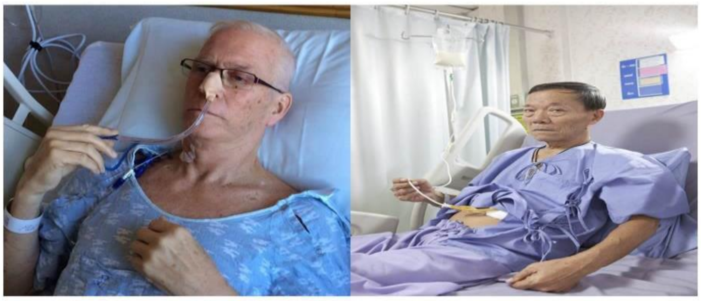

Video of what a procedure would entail?

Video can be accessed at this link [(133) Communicating Health Information Video Scenario V1 29 11 18 2018 - YouTube]

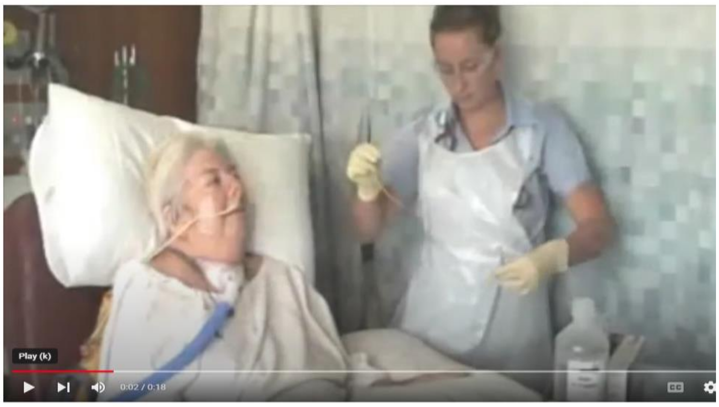

Communicating Health Information Video Scenario V1 29 11 18 2018

Pamphlet on a condition and it's treatment options

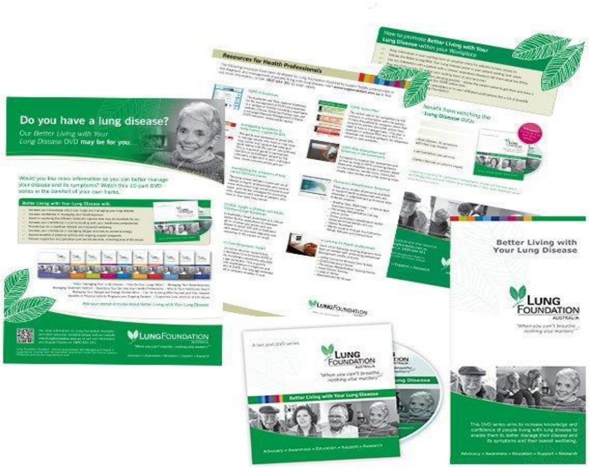

Accessed from the Lung Foundation , Australia. https://lungfoundation.com.au/resources/better-living-with-copd-booklet
